# Retraction: miR-330-5p suppresses glioblastoma cell proliferation and invasiveness through targeting ITGA5

**DOI:** 10.1042/BSR-2017-0019_RET

**Published:** 2021-09-22

**Keywords:** glioblastoma (GBM), ITGA5, miR-330-5p, proliferation

This article is being retracted from *Bioscience Reports* at the request of the Editor-in-Chief and the Editorial Board following receipt of a notification from a reader, alerting the Editorial Board to incorrect data in Figure 4A, as well as a duplicated panel in Figure 3E. Post publication review by the Editorial Office also identified signs of image manipulation in the original versions of Figures 3B, 3C and 3E which were replaced by the authors during the revision process.

The authors have been contacted in regards to the Retraction, and have not responded to the Journal's queries and the concerns raised. Given the extent of the issues raised, the Editorial Board stand by the decision to retract the article.

